# The therapeutic potential of orphan GPCRs, GPR35 and GPR55

**DOI:** 10.3389/fphar.2015.00069

**Published:** 2015-04-15

**Authors:** Derek M. Shore, Patricia H. Reggio

**Affiliations:** Center for Drug Discovery, Department of Chemistry and Biochemistry, University of North Carolina GreensboroGreensboro, NC, USA

**Keywords:** GPR35, CXCR8, GPR55, kynurenic acid, LPI, 2-oleoyl LPA

## Abstract

The G protein-coupled receptor (GPCR) superfamily of integral proteins is the largest family of signal transducers, comprised of ∼1000 members. Considering their prevalence and functional importance, it’s not surprising that ∼60% of drugs target GPCRs. Regardless, there exists a subset of the GPCR superfamily that is largely uncharacterized and poorly understood; specifically, more than 140 GPCRs have unknown endogenous ligands—the so-called *orphan GPCRs*. Orphan GPCRs offer tremendous promise, as they may provide novel therapeutic targets that may be more selective than currently known receptors, resulting in the potential reduction in side effects. In addition, they may provide access to signal transduction pathways currently unknown, allowing for new strategies in drug design. Regardless, orphan GPCRs are an important area of inquiry, as they represent a large gap in our understanding of signal transduction at the cellular level. Here, we focus on the therapeutic potential of two recently deorphanized GPCRs: GPR35/CXCR8 and GPR55. First, GPR35/CXCR8 has been observed in numerous tissues/organ systems, including the gastrointestinal tract, liver, immune system, central nervous system, and cardiovascular system. Not surprisingly, GPR35/CXCR8 has been implicated in numerous pathologies involving these tissues/systems. While several endogenous ligands have been identified, GPR35/CXCR8 has recently been observed to bind the chemokine CXCL17. Second, GPR55 has been observed to be expressed in the central nervous system, adrenal glands, gastrointestinal tract, lung, liver, uterus, bladder, kidney, and bone, as well as, other tissues/organ systems. Likewise, it is not surprising that GPR55 has been implicated in pathologies involving these tissues/systems. GPR55 was initially deorphanized as a cannabinoid receptor and this receptor does bind many cannabinoid compounds. However, the GPR55 endogenous ligand has been found to be a non-cannabinoid, lysophophatidylinositol (LPI) and subsequent high throughput assays have identified other GPR55 ligands that are not cannabinoids and do not bind to either the cannabinoid CB1 and CB2 receptors. Here, we review reports that suggest that GPR35/CXCR8 and GPR55 may be promising therapeutic targets, with diverse physiological roles.

## Introduction

The G protein-coupled receptor (GPCR) superfamily of transmembrane-spanning proteins is composed of ∼1,000 members (Lagerstrom and Schioth, 2008) and comprises ∼3% of the human genome (Insel et al., 2007). Considering their ubiquity and central importance to signal transduction, it is not surprising that ∼60% of pharmaceuticals target GPCRs (McCusker et al., 2007). Unfortunately, many of these therapies have numerous side effects, due to a lack of receptor subtype selectivity (Wang and Lewis, 2013) and/or a pathological interference with physiological signaling (Kenakin, 2005).

However, the therapeutic potential of GPCRs is not even close to being exhausted. While GPCRs are the most exploited therapeutic target for drug design, more than 140 GPCRs have unknown endogenous ligands (Levoye et al., 2006); these comprise the so-called *orphan receptors*. Orphan GPCRs offer tremendous promise, as they may provide novel therapeutic targets that may be more selective than currently known receptors, resulting in the potential reduction in side effects. More generally, they may provide access to signal transduction pathways currently unknown, allowing for new strategies in drug design. Regardless of their therapeutic potential, orphan GPCRs are an important area of inquiry, as they represent a large gap in our understanding of signal transduction at the cellular level.

In this review, we focus on two recently deorphanized GPCRs: GPR35/CXCR8 and GPR55. First, GPR35/CXCR8 has been observed in numerous tissues/organ systems, including the gastrointestinal tract, liver, immune system, central nervous system, and cardiovascular system. Not surprisingly, GPR35/CXCR8 has been implicated in numerous pathologies involving these tissues/systems. While several endogenous ligands have been identified (including kynurenic acid and 2-oleoyl lysophosphatidic acid, GPR35/CXCR8 has recently been observed to bind (and signal at nanomolar concentrations) the chemokine CXCL17 (Maravillas-Montero et al., 2015). Second, GPR55 has been observed to be highly expressed in the central nervous system, as well as adrenal glands, gastrointestinal tract, lung, liver, uterus, bladder, kidney, as well as other tissues/organ systems. Likewise, it is not surprising that GPR55 has been implicated in pathologies involving these tissues/systems. GPR55 was initially deorphanized as a cannabinoid receptor. This receptor does bind many cannabinoid compounds. Interestingly, while GPR55 binds multiple cannabinoid ligands, lysophophatidylinositol is currently thought to be its endogenous ligand. However, the GPR55 endogenous ligand has been found to be LPI and subsequent high throughput assays have identified other GPR55 ligands that are not cannabinoids and do not bind to either the cannabinoid CB1 and CB2 receptors. Here, we review reports that suggest that GPR35/CXCR8 and GPR55 may be promising therapeutic targets, with diverse physiological roles.

## GPR35/CXCR8

**FIGURE 1:**
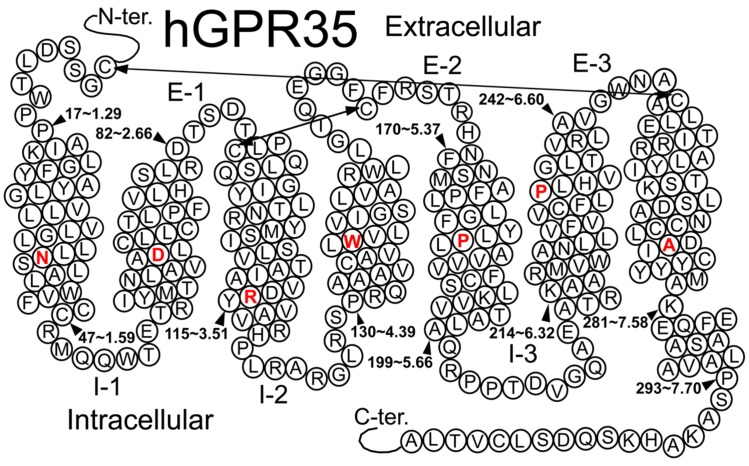
**Helix net representation of human GPR35/CXCR8 receptor structure**. The most highly conserved residue in each transmembrane helix (among Class A GPCRs) is shown in red. Possible disulfide bridges are indicated by double-headed arrows.

GPR35/CXCR8, a recently deorphanized rhodopsin-like, Class A GPCR (Maravillas-Montero et al., 2015), was discovered by O’Dowd et al. (1998) during a human genomic DNA screen (see **Figure 1**). In human, GPR35/CXCR8 is localized to Chromosome 2q37.3 (Genbank accession #: AF027957; O’Dowd et al., 1998). Currently, it is thought that GPR35/CXCR8 is most homologous with the purinergic receptor GPR23/P2Y9 (∼32% overall sequence identity; O’Dowd et al., 1998), the nicotinic acid receptor HM74 (∼30% overall sequence identity; O’Dowd et al., 1998), as well as GPR55 (∼27% overall sequence identity; Sawzdargo et al., 1999). GPR35/CXCR8 was originally reported to consists of 309 amino acids (O’Dowd et al., 1998); however, in Okumura et al. (2004) a splice variant of GPR35/CXCR8 was discovered (i.e., GPR35b) that has an N-terminal expansion of 31 amino acids. GPR35b was discovered from a cDNA library produced from human gastric cancer cells (Okumura et al., 2004).

## GPR35/CXCR8 Pharmacology

**FIGURE 2:**
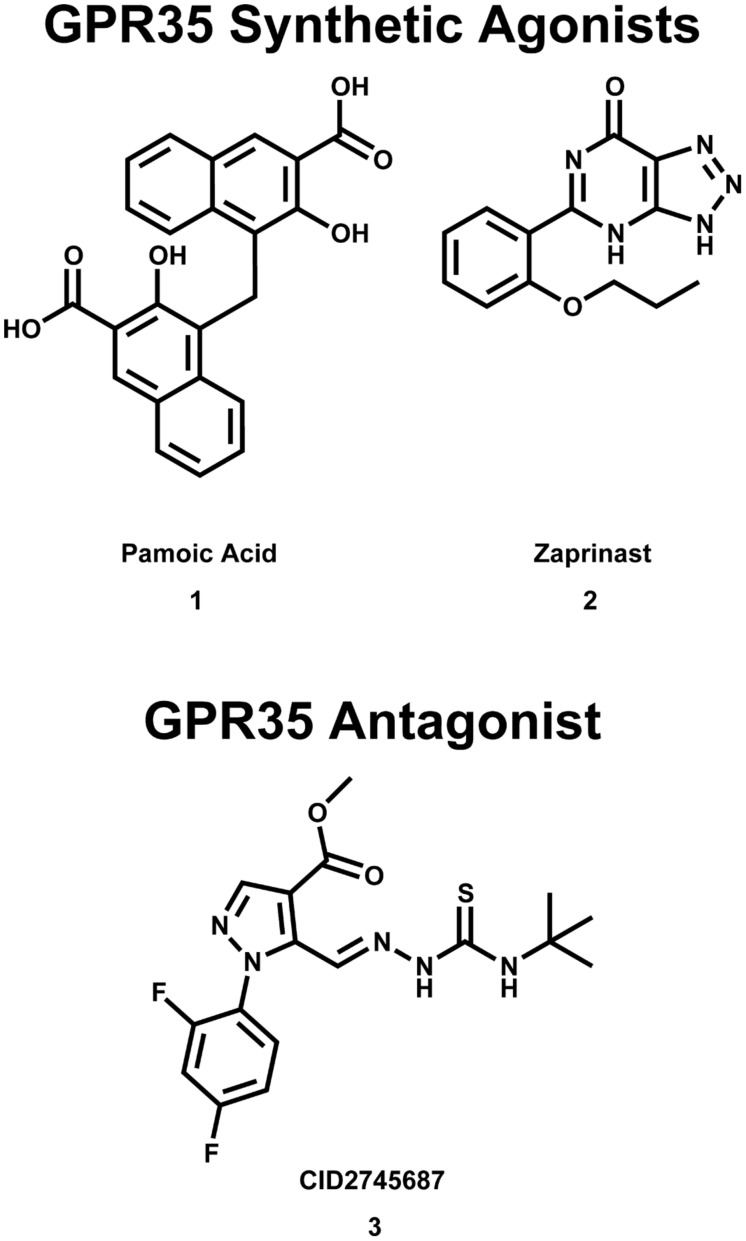
**Synthetic GPR35/CXCR8 agonists and antagonists**. These compounds are among the best characterized synthetic compounds at GPR35.

Early reports suggested that GPR35/CXCR8 may signal via G_i/o_ proteins; specifically, promiscuous (e.g., G_α16_) and chimeric G proteins (e.g., G_qs5_, G_qo5_, G_qi9_, etc.) were used to aid the detection of GPR35/CXCR8 signal transduction (Taniguchi et al., 2006; Wang et al., 2006). The use of these chimeric G proteins enables GPCRs that preferentially couple G_i/o_ (and/or G_s_) to couple to G_q_; allowing receptor activation to be observed as changes in intracellular (IC) [Ca^2+^] (Conklin et al., 1993; Milligan et al., 1996; Coward et al., 1999). Using this methodology, kynurenic acid (**12**) and zaprinast (**2**) (see **Figures 2 and 4**) were the first agonists identified for GPR35/CXCR8 (Taniguchi et al., 2006; Wang et al., 2006). It was also reported that kynurenic acid (**12**) stimulated [^35^S]GTPγS binding in CHO cells expressing human GPR35/CXCR8 and that this stimulation was blocked by pretreatment of pertussis toxin, suggesting the involvement of G_i/o_ proteins (Wang et al., 2006). Consistently, it has been reported that kynurenic acid (**12**) and zaprinast (**2**) act as agonists of heterologously expressed GPR35/CXCR8 in rat sympathetic neurons with endogenous G proteins (Guo et al., 2008). The authors reported that kynurenic acid (**12**) and zaprinast’s (**2**) agonism of GPR35/CXCR8 resulted in the inhibition of N-type calcium channels; this inhibition was blocked by pertussis toxin pretreatment, again suggesting the involvement of G_i/o_ proteins (Guo et al., 2008). Furthermore, it has been reported that agonism of GPR35/CXCR8 by kynurenic acid (**12**) and zaprinast (**2**) resulted in a signification reduction in interleukin (IL)-4 release from α-galactosylceramide-activated human invariant natural killer T (iNKT) cells, and that this reduction was abolished by pre-treatment with pertussis toxin (Fallarini et al., 2010). Interestingly, it has been reported that GPR35/CXCR8 may also couple to G_α13_ (Jenkins et al., 2010, 2011). Together, these results suggest that GPR35/CXCR8 couples to G_α13_ and G_i/o_ proteins.

It has also been reported that pamoic acid (**1**) (see **Figure 2**) acts as an agonist of GPR35b (Zhao et al., 2010). Pamoic acid (**1**) was discovered to be an agonist of GPR35b during a screen of the Prestwick Chemical Library (Zhao et al., 2010). It was observed that activation of mouse GPR35b by pamoic acid (**1**) resulted in the recruitment of β-arrestin 2-green fluorescent protein and receptor internalization in U2OS cells (Zhao et al., 2010). Agonism of GPR35b by pamoic acid (**1**) also resulted in the activation of ERK1/2 (Zhao et al., 2010). Additionally, the pamoic acid (**1**)-mediated recruitment of β-arrestin 2 and activation of ERK1/2 was blocked by CID2745687 (**3**)—a GPR35/CXCR8 antagonist (see **Figure 2**; Zhao et al., 2010). The pamoic acid (**1**)-mediated recruitment of β-arrestin 2 and activation of ERK1/2 was also blocked by pre-treatment with pertussis toxin (Zhao et al., 2010). Coupling between GPR35/CXCR8 and β-arrestin 2 has also been observed by groups (Jenkins et al., 2010, 2011), who also employed a receptor-β-arrestin 2 interaction assay to screen the Prestwick Chemical Library for human and/or rat GPR35/CXCR8 agonists (Jenkins et al., 2010). It has been reported that agonism of GPR35/CXCR8 reduces Ca^2+^ transients in mouse astrocytes (Berlinguer-Palmini et al., 2013). Here, the authors observed that activation of GPR35/CXCR8 by kynurenic acid (**12**) reduced synaptic activity at CA3-CA1 synapses (Berlinguer-Palmini et al., 2013). Altogether, these results suggest that, in addition to coupling to G_α13_ and G_i/o_ proteins, GPR35/CXCR8 activation may result in receptor coupling to β-arrestin 2, receptor internalization, ERK1/2 activation, as well as impacting Ca^2+^ transients and synaptic activity.

## GPR35/CXCR8 Expression Profile and Therapeutic Potential

### GPR35/CXCR8 Gastrointestinal Tract and Liver Expression/Therapeutic Potential

First, Northern blot analysis was used to observe GPR35/CXCR8 expression in rat small intestine (O’Dowd et al., 1998). As already mentioned, GPR35b was also detected during a screen of a cDNA library produced from human gastric cancer cells (Okumura et al., 2004). Likewise, it has been reported that that human GPR35/CXCR8 is most highly expressed in the small intestine and is also highly expressed in the colon and stomach (Wang et al., 2006; Imielinski et al., 2009). While it has been reported that mouse GPR35/CXCR8 is most highly expressed in the spleen, GPR35/CXCR8 expression has also been observed in mouse small intestine, colon, and stomach (Wang et al., 2006). Analogously, it has been reported that rat GPR35/CXCR8 is expressed in rat stomach, small-intestine, and colon (Taniguchi et al., 2006). Interestingly, GPR35/CXCR8 expression has also been observed in HT-29, a human colon cancer cell line (Deng et al., 2011). Finally, GPR35/CXCR8 expression has been observed in embryonic mouse rectum (Hilger et al., 2013).

Modest levels of GPR35/CXCR8 expression has also been observed in both human, mouse, and rat liver (Taniguchi et al., 2006; Wang et al., 2006). Consistently, GPR35/CXCR8 expression has been observed in embryonic mouse liver (Hilger et al., 2013). As described later, the modest level of GPR35CXCR8 expression in liver does not necessarily preclude physiologically important function.

Considering GPR35/CXCR8’s high levels of expression in human small intestine, colon, and stomach, it’s not surprising that many reports have implicated GPR35/CXCR8 in numerous pathologies of the gastrointestinal tract. As already mentioned, the splice variant GPR35b was discovered from a cDNA library produced from human gastric cancer cells (Okumura et al., 2004); here, the authors reported results that suggest that GPR35b is involved in the transformation of NIH3T3 cells. In addition, it was reported that GPR35b’s expression was up-regulated in gastric cancer tissues (Okumura et al., 2004). As already mentioned, GPR35/CXCR8 expression has been observed in HT-29 cells (a human colon cancer cell line; Deng et al., 2011). Together, these results suggest that GPR35/CXCR8 may be an attractive target in the development of new gastric cancer treatments.

**FIGURE 3:**
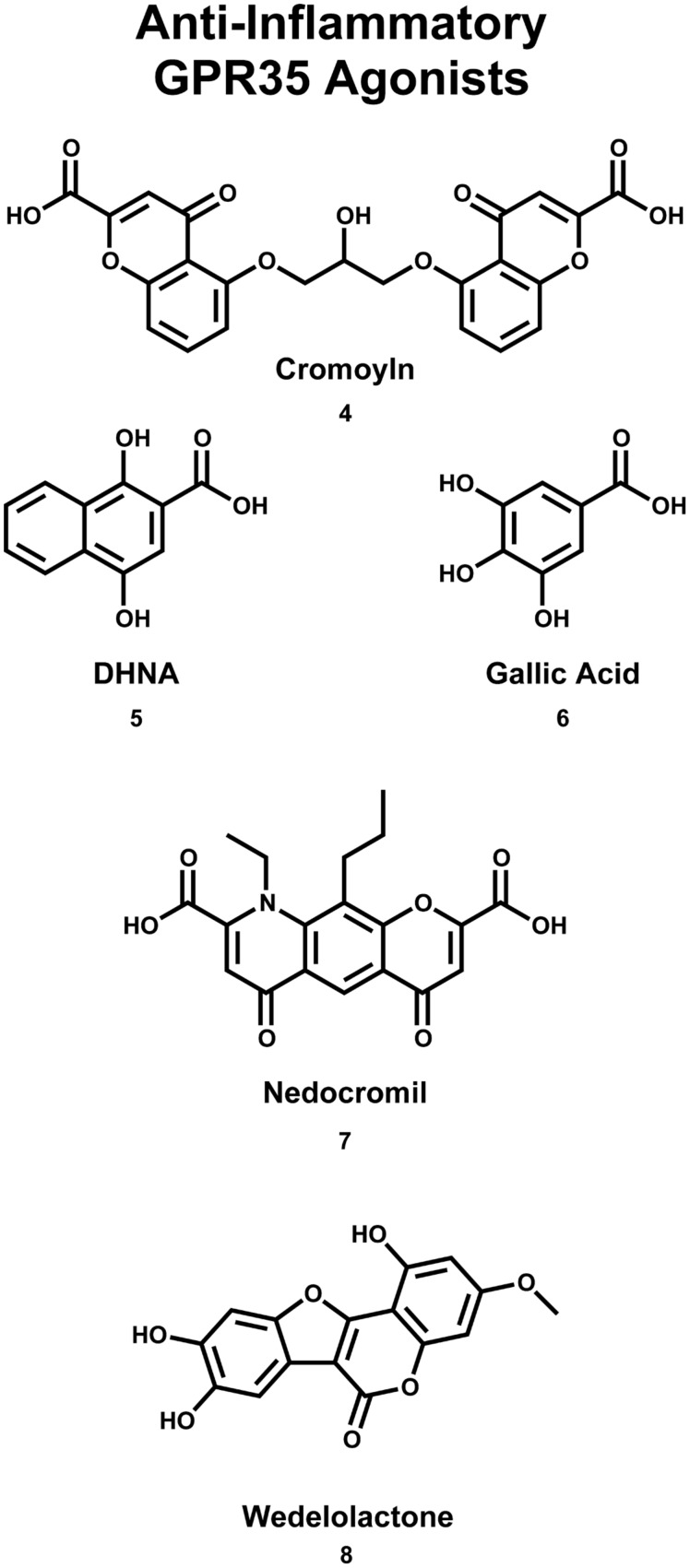
**Compounds that are used (or have the potential) to treat asthma/inflammation**. Cromoyln (4) and Nedocromil (7) are used in the treatment of asthma/inflammatory states. Gallic acid (6) and wedelolactone (8) have been reported to be potential anti-asthma/anti-inflammatory compounds. DHNA (5) has been suggested that it may be useful in the treatment of bowel inflammation.

GPR35/CXCR8 has also been implicated in early onset inflammatory bowel disease (IBD; Imielinski et al., 2009). Here, the authors reported the results of a genome-wide association study (GWAS), which identified a signification association between the chromosome region 2q37 (containing GPR35/CXCR8) and early-onset IBD (Imielinski et al., 2009). Interestingly, GPR35/CXCR8 has been implicated in both primary sclerosing cholangitis (PSC) and ulcerative colitis (UC; Ellinghaus et al., 2013). Specifically, the authors reported the results of a GWAS, which identified a missense single nucleotide polymorphism (SNP), GPR35/CXCR8 rs3749171, that results in a shift from a threonine to a methionine (T3.44(108)M, using Ballesteros–Weinstein nomenclature; Ellinghaus et al., 2013). This polymorphism of GPR35/CXCR8 was associated with both PSC and UC (Ellinghaus et al., 2013). The authors also reported that their structural modeling results suggest that this polymorphism is located in transmembrane helix (TMH) 3 (Ellinghaus et al., 2013); this may suggests that the polymorphism impacts GPR35/CXCR8’s ability to activate. While UC is a form of IBD, PSC is a hepatic disease, involving inflammation of bile ducts inside and outside of the liver. This association between the GPR35/CXCR8 polymorphism and PCS may be surprising, given the modest level of GPR35/CXCR8 expression in liver. However, this association is more easily understood if one considers that the most common comorbidity of PSC is IBD, where 60–80% of PSC patients of Northern European descent also reported IBD (Karlsen et al., 2010). This high degree of comorbidity may suggests an indirect relationship between PSC and GPR35/CXCR8 that is not yet fully understood. Recently, the results of another GWAS and ImmunoChip single-nucleotide polymorphism screening have been reported (Yang et al., 2015). Here, the authors determined an association between polymorphisms at 2q37 (in a Korean patients) and Crohn’s disease (i.e., an intractable IBD; Yang et al., 2015). Together, these reports suggest that GPR35/CXCR8 may be involved in IBD, for both Eastern and Western populations. Encouragingly, GPR35/CXCR8’s therapeutic potential to treat these pathologies is already being explored. For example, it has been reported that the GPR35/CXCR8 agonist 1,4-dihydroxy-2-naphthoic acid [DHNA (**5**), see **Figure 3**] may be effective in treating bowel inflammation (Okada et al., 2006).

### GPR35/CXCR8 Innate Immune System Expression/Therapeutic Potential

GPR35/CXCR8 has been reported to be expressed in numerous types of immune cells/tissues. High levels of human GPR35/CXCR8 expression has been observed in the spleen, fetal spleen, peripheral leukocytes, and modest expression has been observed in the thymus, monocytes, T cells, natural killer cells, neutrophils, eosinophils, and dendritic cells (Wang et al., 2006). In addition, human GPR35/CXCR8 expression has also been observed in peripheral monocytes (Barth et al., 2009), primary macrophages (Sparfel et al., 2010), as well as in mast cells, basophils, and eosinophils (Yang et al., 2010). Human GPR35/CXCR8 expression has also been observed in iNKT cells (Fallarini et al., 2010). Finally, human GPR35/CXCR8 expression has also been observed in CXCL17-responsive monocytes, and the THP-1 monocytoid cell line (Maravillas-Montero et al., 2015).

**FIGURE 4:**
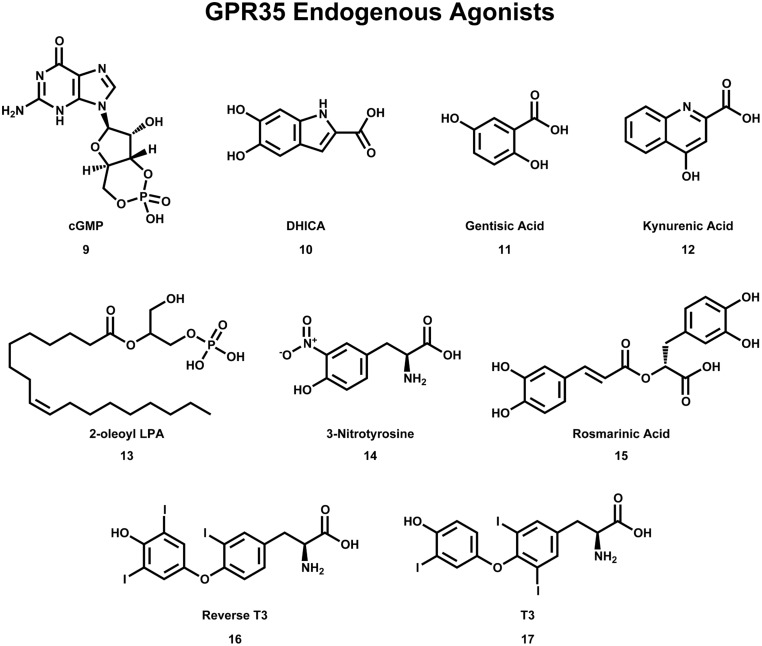
**Compounds that have been reported to be endogenous agonists of GPR35/CXCR8**.

Considering GPR35/CXCR8’s high levels of expression in immune cells, tissues, and organs, it’s not surprising that several reports have implicated GPR35/CXCR8’s functional importance to immune system function and pathologies. For example, it has been reported that GPR35/CXCR8 may participate in the firm arrest/adhesion of leukocytes to vascular endothelium (Barth et al., 2009). The authors, using a vascular flow model, reported that kynurenic acid (**12**) (a GPR35/CXCR8 agonist, see **Figure 4**) induced the firm arrest of monocytes to both fibronectin and ICAM-1. The arrest of monocytes to fibronectin and ICAM-1 was reported to be mediated by β_1_- and β_2_-integrin, respectively (Barth et al., 2009). The kynurenic acid (**12**) inducement of firm arrest was significantly reduced by pre-treatment with pertussis toxin; this observation suggests that this process is G_i/o_-mediated, consistent with the hypothesized involvement of GPR35/CXCR8. Furthermore, the authors reported that the kynurenic acid (**12**) inducement of firm arrest was also significantly reduced by the use of short hairpin RNA silencing of GPR35/CXCR8. The authors also reported that kynurenic acid (**12**) induced firm adhesion of neutrophils to an ICAM-1 expressing monolayer and induced neutrophil shedding of surface L-selectin. Altogether, these results suggest that GPR35/CXCR8 may be an important participant in leukocyte recruitment.

Additionally, GPR35/CXCR8 has been suggested to be an important target in the treatment of asthma (Yang et al., 2010). First, as already mentioned, GPR35/CXCR8 expression has been observed in human mast cells (Yang et al., 2010). By binding antigen-specific IgE antibodies at their surface, mast cells are capable of responding to allergens (Kikuchi et al., 2002). Upon IgE antibody-binding, mast cells release histamine (Kikuchi et al., 2002). Thus, mass cell stabilizers bind the IgE receptor on plasma membrane of mast cells (blocking the binding of IgE antibodies), effectively preventing the release of histamine; one use of these compounds is to treat asthma (MacGlashan, 2008). Interestingly, the results of calcium flux and inositol phosphate accumulation assays suggest that the asthma medications cromolyn disodium (**4**) and nedocromil sodium (**7**) (see **Figure 3**) are agonists of GPR35/CXCR8 (Yang et al., 2010). The authors also reported that GPR35/CXCR8 mRNA is upregulated upon the introduction of IgE antibodies—suggesting GPR35/CXCR8 is involved in the mast cell allergen response (Yang et al., 2010). It has also been reported that the anti-inflammatory compounds, gallic acid (**6**) and wedelolactone (**8**) (see **Figure 3**), are agonists of GPR35/CXCR8, in cells that endogenously express GPR35/CXCR8 (e.g., HT-29 cells), as well as the engineered U2OS cell line (Deng and Fang, 2012). Altogether, these results suggest that GPR35/CXCR8 may be an important target in the development of novel asthma and anti-inflammatory medications; for additional review of GPR35/CXCR8 and its relationship to inflammation, see (MacKenzie et al., 2011).

### GPR35/CXCR8 Nervous System Expression/Therapeutic Potential

GPR35/CXCR8 has been reported to be expressed within rat and mouse nervous systems. High levels of GPR35/CXCR8 expression have been observed in rat dorsal root ganglion (DRG; Taniguchi et al., 2006; Ohshiro et al., 2008), whereas modest expression was observed in rat brain (general), cerebrum, and spinal cord (Taniguchi et al., 2006). It has also been reported that endogenously expressed GPR35/CXCR8 in DRG are functional (Ohshiro et al., 2008). In cAMP assays, both kynurenic acid (**12**) and zaprinast (**2**) appeared as agonist at GPR35/CXCR8-expressing DRG and this agonism was blocked by pre-treatment with pertussis toxin (Ohshiro et al., 2008). Recently, GPR35/CXCR8 expression has been observed in the CA1 field of rat hippocampus (Alkondon et al., 2014). The authors also measured the frequency of spontaneous action potentials in rat hippocampal slices in the presence of several GPR35/CXCR8 agonists; reported results suggest that the detected CA1 field GPR35/CXCR8 are functional and serve to suppress neuronal activity (Alkondon et al., 2014). Analogous to rat GPR35/CXCR8 expression, mouse GPR35/CXCR8 expression has been observed in dorsal root ganglia (Cosi et al., 2011; Berlinguer-Palmini et al., 2013), spinal cord (Cosi et al., 2011), as well as in cultured astrocytes (Berlinguer-Palmini et al., 2013) and glial cells (Cosi et al., 2011). GPR35/CXCR8 expression has also been observed in embryonic mouse corpus striatum mediale and hypothalamus (Hilger et al., 2013).

Considering GPR35/CXCR8’s expression within the rat and mouse nervous systems, it’s not surprising that many reports have implicated GPR35/CXCR8 in mild mental retardation. Specifically, the results of a GWAS suggests an association between terminal deletions on chromosome 2q37.3 and mild retardation/a phenotype that resembles Albright hereditary osteodystrophy (AHO)/pseudopseudohypoparathyroidism (Shrimpton et al., 2004). The authors observed that patients with AHO had a deletion of either the maternal or paternal chromosome 2q37.3 (i.e., the chromosomal location of GPR35/CXCR8). However, as MacKenzie et al. (2011) have correctly mentioned, these deletions were not directly mapped to GPR35/CXCR8, but rather a 3Mb region; this region contains at least 30 other genes that may contribute to the AHO phenotype. Shrimpton et al. (2004) acknowledge this difficulty, but hypothesize that the gene that encodes GPR35/CXCR8 is the most likely candidate within the 3 Mb region; this is because it is known that classical AHO results from a deletion/mutation of the alpha subunit of GNAS1 gene (chromosomal location: 20q13.2; Rao et al., 1991). GNAS1 encodes for the alpha subunit of the G_s_ protein. Therefore, Shrimpton et al. (2004) hypothesized that if mutation/deletion of the G_s_ protein results in AHO, then perhaps mutation/deletion of a GPCR (e.g., GPR35/CXCR8) that activates G_s_ would also result in AHO. However, since Shrimpton et al. (2004) report, several groups have reported that GPR35/CXCR8 couples to G_α13_ and G_i/o_ proteins (as described earlier); to our knowledge, there are no known agonists of GPR35/CXCR8 that induce activation of G_s_. This observation certainly does not rule out GPR35/CXCR8’s involvement in AHO, but it does suggests that it is not through a GPR35/CXCR8-G_s_ mechanism.

Additionally, it has been suggested that GPR35/CXCR8 may be an attractive anti-nociception target. As already mentioned, it has been reported that functional GPR35/CXCR8 is expressed in rat nociceptive DRG neurons (Ohshiro et al., 2008). As expected, these DRG neurons were reported to also expressed TRPV1 receptors; DRG neurons that express TRPV1 receptors have been reported to mediate hyperalgesia, neurogenic inflammation, and neuropathic pain (Lawson, 2002). Interestingly, it was also observed that GPR35/CXCR8 and TRPV1 co-localized in rat DRG neurons (Ohshiro et al., 2008). Thus, based on the observations that (1) GPR35/CXCR8 is expressed in rat nociceptive DRG neurons, and (2) GPR35/CXCR8 co-localizes with TRPV1 receptors in these DRG neurons, the authors hypothesized that GPR35/CXCR8 may be involved nociception (Ohshiro et al., 2008). Consistent with this hypothesis, it has been reported that pamoic acid (**1**) (a GPR35/CXCR8 agonist, see **Figure 2**) produced dose-dependent antinociception in mice (Zhao et al., 2010). Specifically, using an abdominal constriction test of visceral pain (in mice), the authors reported that a dose of 40.5 mg/kg pamoic acid (**1**) resulted in 50% antinociception, whereas a dose of 100 mg/kg pamoic acid (**1**) resulted in essentially complete antinociception (Zhao et al., 2010). Other groups have also reported that both kynurenic acid (**12**) and zaprinast (**2**) (GPR35/CXCR8 agonists, see **Figures 2** and 4) also produce antinociception in mice (Cosi et al., 2011). Using an acetic acid writhing test (in mice), the authors reported that a dose of 100 mg/kg L-kynurenine [a metabolic precursor of kynurenic acid (**12**), which more easily crosses the blood brain] decreased the number of writhes by 29%, whereas a dose of 300 mg/kg reduced writhes by 58%. Consistent with its reported superior *in vitro* efficacy (i.e., Ca^2+^ mobilization assays using a G_αqi5_ chimera) at both human and rat (Taniguchi et al., 2006; Wang et al., 2006), zaprinast (**2**) administration resulted in anti-nociception at lower doses than kynurenic acid (**12**) (Cosi et al., 2011). The authors reported that a dose of 5 mg/kg zaprinast (**2**) decreased the number of writhes by 58% (Cosi et al., 2011); interestingly, zaprinast-induced anti-nociception did not appear dose-dependent at the doses tested (Cosi et al., 2011). The authors also reported that administering the maximal effective doses of kynurenic acid (**12**) and zaprinast (**2**) concurrently did not result in additional anti-nociception (i.e., the resultant analgesia was not additive); this may suggests that kynurenic acid (**12**) and zaprinast (**2**) are acting at the same target (i.e., GPR35/CXCR8) and have saturated available receptors (Cosi et al., 2011).

Subsequently, an indirect relationship between GPR35/CXCR8 and antinociception has been suggested. It has been reported that Ret tyrosine kinase receptor (Ret) knockout mice experienced cold and mechanical hyperalgesia (Franck et al., 2011). Signaling via Ret is one of the mechanisms that impacts the development of sensory neurons, and serves an important role in regulating many ion channels and receptors (e.g., Nav1.8, Nav1.9, ASIC2a, P2X3, TrpC3, TrpM8, TrpA1, delta opiod receptor, MrgD, MrgA1, and MrgB4; Franck et al., 2011). The authors investigated GPR35/CXCR8 regulation in Ret-knockout mice, due to its co-expression with TRPV1 (Ohshiro et al., 2008); the authors observed that knocking out Ret resulted in a complete loss of GPR35/CXCR8 expression (Franck et al., 2011). Consequently, the authors hypothesized that Ret may regulate cold and mechanical sensitivity/analgesia via modulation of GPR35/CXCR8 (Franck et al., 2011). While the authors’ observations do not prove GPR35/CXCR8’s involvement in nociception, they are consistent with prior reports that also suggested a relationship between GPR35/CXCR8 and pain. These reports suggest that GPR35/CXCR8 may be a promising anti-nociception target that warrants additional inquiry.

### GPR35/CXCR8 Cardiovascular System Expression/Therapeutic Potential

GPR35/CXCR8 has been reported to be expressed within rat and mouse cardiovascular systems. first, modest GPR35/CXCR8 expression has been observed in rat heart (Taniguchi et al., 2006). Second, GPR35/CXCR8 expression has been observed in neonatal mouse cardiac myocytes and HL-1 cardiomyocytes (Ronkainen et al., 2014).

Despite the limited evidence of GPR35/CXCR8 cardiovascular system expression, there are reports that suggest GPR35/CXCR8 may play a role in cardiovascular disease. First, a GWAS (that employed 2 machine learning algorithms, Random Forest and RuleFit) was used to identify a non-synonymous SNP (GPR35/CXCR8 rs3749172), which results in a shift from a serine to an arginine (S294R, on the C-terminus; Sun et al., 2008). This polymorphism of GPR35/CXCR8 was associated with higher coronary artery calcification (CAC) burden (Sun et al., 2008). CAC is measure of subclinical coronary atherosclerotic calcified plaque (Sun et al., 2008) and has been used to predict coronary artery disease events, in both asymptomatic (Arad et al., 2000) and symptomatic adults (Keelan et al., 2001). While GPR35/CXCR8’s role in CAC is unclear, Sun et al. (2008) hypothesized that this polymorphism (which results in C-terminus residue switch) may alter receptor phosphorylation, ultimately impacting receptor coupling with effector proteins (e.g., G proteins, arrestins, etc.; Sun et al., 2008); this hypothesis has yet to be tested.

It has also been suggested that there is a relationship between GPR35/CXCR8 and heart disease (Min et al., 2010). Specifically, the authors performed expression microarray analyses on 14 patients (12 heart failure patients and 2 healthy patients) and constructed datasets to identify relationships between genes expression levels and clinical parameters (e.g., pulmonary artery pressure, left ventricular ejection fraction, and brain natriuretic peptide mRNA level; Min et al., 2010). Interestingly, the authors identified GPR35/CXCR8 as a highly expressed in heart failure patients (Min et al., 2010). The authors also reported that GPR35/CXCR8 over-expression (in neonatal rat cardiomyocytes) resulted in hypertrophy (Min et al., 2010). Lastly, the authors reported that GPR35/CXCR8 knock-out mice were measured to have a 37.5 mmHG increase in blood pressure as compared to WT mice (Min et al., 2010).

Recently, it has been reported that GPR35/CXCR8 expression (in a mouse model of progressive cardiac hypertrophy) is an early marker of heart failure and a marker for cardiac hypoxia in acute myocardial infarction (MI; Ronkainen et al., 2014). The authors observed that hypoxia increased GPR35/CXCR8 expression in mouse neonatal mouse cardiomyocytes and HL-1 cells. In the neonatal cardiomyocytes, this increase in GPR35/CXCR8 expression was statistically significant by 12 h (post hypoxia), and was continuing to increase at 48 h—the last time point measured. The authors also observed that hypoxia increased GPR35/CXCR8 expression in embryonic cardiomyocytes, suggesting that the increase in GPR35/CXCR8 expression is not developmentally regulated. Furthermore, the authors reported that this increase in GPR35/CXCR8 expression did indeed result in a significant increase in GPR35/CXCR8 receptors at the cell surface. The authors also reported that this hypoxia-induced increase in GPR35/CXCR8 expression is mediated by hypoxia-inducible factor 1 (HIF-1; a member of a family of transcription factors that respond to changes in oxygen concentration). Overexpression of GPR35/CXCR8 (in GPR35/CXCR8-Venus-expressing neonatal mouse cardiomyocytes) influenced cell morphology, resulting in pronounced cellular ruﬄing and the formation of retraction fibers (however, cell size was not impacted; Ronkainen et al., 2014). In addition, the authors observed that GPR35/CXCR8 expression increased in mouse models of acute MI; to model acute MI, the rodent’s left anterior descending (LAD) artery was ligated. GPR35/CXCR8 expression was significantly increased 1 day after the operation, but returned to SHAM-operated mice levels after 4 days (Ronkainen et al., 2014). The authors also measured GPR35/CXCR8 expression in mouse models of pressure-load induced cardiac hypertrophy; to model, the mice were subjected to transversal aortic constriction (TAC). Here, the authors observed that mouse GPR35/CXCR8 expression was significantly increases 2 weeks after TAC operation, and remained elevated at 4 weeks post TAC operation. Altogether, these reports may suggest that GPR35/CXCR8 may be an important target in the development of novel cardiovascular disease therapies and may act as an early marker of cardiac pathologies.

### Additional GPR35/CXCR8 Expression/Therapeutic Opportunities

GPR35/CXCR8 has also been reported to be expressed within several additional tissues/organs, in which the therapeutic potential has not yet been as thoroughly explored. For example, GPR35/CXCR8 expression has been observed in human and mouse adipose, kidney, lung, and pancreas (Wang et al., 2006). In addition, GPR35/CXCR8 expression has been observed in human skin (Yang et al., 2012). Mouse GPR35/CXCR8 expression has been observed in embryonic lung, as well as in genital tubercle and tooth bud (Hilger et al., 2013). Finally, rat GPR35/CXCR8 expression was observed in lung, as well as bladder, skeletal muscle, and uterus (Taniguchi et al., 2006).

GPR35/CXCR8 has also been implicated in at least two additional pathologies. First, the results of GWAS have identified GPR35/CXCR8 SNP that have been associated with diabetes (Horikawa et al., 2000; Vander Molen et al., 2005); for review, see (MacKenzie et al., 2011). Second, the results of an array-based molecular karyotyping study identified three possible *de novo* copy number variations (CNVs; chromosomal regions 1q41, 2q37.3, and 8q24.3) that were present in 3 of 47 patients with either VATER/VACTERL association (41 patients) or VATER/VACTERL-phenotype (six patients; Hilger et al., 2013). VATER/VACTERL association refers to the non-random co-occurrence of at least three congenital anomalies: vertebral defects, anorectal malformations, cardiac defects, tracheoesophageal fistula with or without esophageal atresia, renal malformations, and limb defects; VATER/VACTERL-phenotype refers to the non-random co-occurrence of at least two of these congenital anomalies (Hilger et al., 2013). The three possible *de novo* CNV aberrations were confirmed using qPCR (Hilger et al., 2013). The authors used the Mouse Genome Informatics database (consisting of expression and targeted deletions data) to decide which genes (within the three chromosomal regions) to further characterize. GPR35/CXCR8 was one of the genes selected for additional characterization, based on its expression in mouse, genital tubercle, the mesonephros, and rectum (Hilger et al., 2013). However, the authors reported that GPR35/CXCR8 sequence analysis of 192 patients with VATER/VACTERL association/phenotype did not identify any disease-causing mutations. The authors suggest this may be because the sequence analysis may have missed mutations in non-protein-coding exons, or the promoter region; additionally, the authors suggest that the number of patients sequenced may have been too small to detect rare causal mutations. Regardless, due to GPR35/CXCR8’s expression pattern in mice (which aligns with VATER/VACTERL affected tissues/organs), additional inquiry into GPR35/CXCR8’s involvement in this pathology is warranted.

## GPR35/CXCR8 Endogenous Ligands and Deorphanization

As with many orphan GPCRs, there has been some controversy as to what the endogenous ligand(s) is/are for GPR35/CXCR8. The determination of GPR35/CXCR8’s endogenous ligands has been complicated by questions of species selectivity, concentration of specific ligands in various tissue types, as well as concerns regarding assay bias. Here, we will briefly discuss the two best-characterized endogenous ligands of GPR35/CXCR8 [e.g., kynurenic acid (**12**) and lysophosphatidic acid; see **Figure 4**], followed by a discussion of more recently discovered endogenous ligands.

Kynurenic acid (**12**), a metabolite of tryptophan, was the first endogenous ligand discovered for GPR35/CXCR8 (Wang et al., 2006). Numerous reports suggests that kynurenic acid (**12**) induces GPR35/CXCR8 signaling via G_i/o_ and G_α13_ proteins (Wang et al., 2006; Guo et al., 2008; Fallarini et al., 2010; Berlinguer-Palmini et al., 2013), as well as β-arrestin 2 and ERK1/2 (Zhao et al., 2010; Jenkins et al., 2011). Interestingly, kynurenic acid (**12**) is present in many tissues that express GPR35/CXCR8 (e.g., brain, colon, intestine, kidney, lung, muscle, pancreas, and spleen; MacKenzie et al., 2011). Thus, due to its endogenous activation of GPR35/CXCR8, as well as being present in many of the same tissues, kynurenic acid (**12**) has been suggested to be ‘the’ endogenous ligand of GPR35/CXCR8. However, at least two major concerns regarding this designation have been raised; first, micromolar concentrations of kynurenic acid (**12**) are required to activate GPR35/CXCR8 (Wang et al., 2006), with some groups reporting almost no response even at very high concentrations (Oka et al., 2010b). Second, it has been reported that kynurenic acid (**12**) is 40–100 fold more potent at rat than human (Barth et al., 2009; Jenkins et al., 2011), potentially suggesting that kynurenic acid (**12**) may be more likely to be the endogenous ligand of rat GPR35/CXCR8. For additional review of kynurenic acid (**12**) and GPR35/CXCR8, see (MacKenzie et al., 2011; Zhao and Abood, 2013).

The second reported endogenous ligand for GPR35/CXCR8 was 2-oleoyl lysophosphatidic acid [2-oleoyl LPA (**13**)]; **Figure 4** (Oka et al., 2010b). 2-oleoyl LPA (**13**) has been reported to be present in serum, plasma, and brain (Sugiura et al., 1999; Noguchi et al., 2009). Despite a less obvious overlap between tissues that express GPR35/CXCR8 and contain 2-oleoyl LPA (**13**), this ligand has been reported to activate human GPR35/CXCR8 with high potency—unlike kynurenic acid (**12**) (Oka et al., 2010b). However, both kynurenic acid (**12**) and 2-oleoyl LPA (**13**) (and LPAs in general) are known to have numerous cellular effects, as well as bind to many different targets (MacKenzie et al., 2011; Zhao and Abood, 2013). This promiscuity may suggests that neither kynurenic acid (**12**) nor 2-oleoyl LPA (**13**) are ‘the’ endogenous ligand for GPR35/CXCR8—rather, GPR35/CXCR8 more likely binds several endogenous ligands and that this is highly species/tissue dependent. For additional review of LPAs and GPR35/CXCR8, see (MacKenzie et al., 2011; Zhao and Abood, 2013).

Consistent with this hypothesis, several other endogenous ligands of GPR35/CXCR8 have been reported. First, several tyrosine metabolites (including 5,6-dihydroxyindole-2-carboxylic acid [DHICA (**10**)], 3,3′,5′-triiodothyronine [reverse T3 (**16**)], 3,3′,5-triiodothyronine [T3 (**17**)], gentisatic acid (**11**), rosmarinic acid (**15**), and 3-nitrotyrosine (**14**), see **Figure 4**) have been reported to act as endogenous ligands of GPR35/CXCR8 (Deng et al., 2012). Interestingly, all of these compounds [with the exception of 3-nitrotyrosine (**14**)] were reported to be more potent at GPR35/CXCR8 than kynurenic acid (**12**) (Deng et al., 2012). Second, the results of a 10,500-ligand PathHunter screen suggest that guanosine-3′,5′-cyclic monophosphate [cGMP (**9**), see **Figure 4**] may activate GPR35/CXCR8, though high micromolar concentrations are required (Southern et al., 2013). Finally, it has recently been reported that the mucosal chemokine CXCL17 activates GPR35/CXCR8 (Maravillas-Montero et al., 2015). CXCL17 was the last chemokine to be described (Pisabarro et al., 2006) and currently is only known to signal via GPR35/CXCR8 (Maravillas-Montero et al., 2015). The authors report that CXC17 activates GPR35/CXCR8 at nanomolar concentrations (in calcium flux assays)—making it significantly more potent at GPR35/CXCR8 than kynurenic acid (**12**) or LPA (Maravillas-Montero et al., 2015). Given CXCL17’s apparent selectivity for GPR35/CXCR8, its potency, and its presence in GPR35/CXCR8-expressing tissues, the authors suggest GPR35 be named CXCR8 (Maravillas-Montero et al., 2015). Here, we adopt this nomenclature; however, the importance of other GPR35/CXCR8 endogenous ligands should not be disregarded.

## GPR35/CXCR8 Structure and Modeling

### Sequence Analysis and Motifs

Several Class A GPCRs have been crystallized, though these solved structures represent only a small fraction of all GPCRs. These crystal structures reveal a common topology that includes seven transmembrane alpha helices (TMHs) that are connected with three intra- and three extracellular (EC) loops; GPCRs also contain an EC N-terminus and an IC C-terminus that begins with a short helical segment (Helix 8) oriented parallel to the membrane surface. To date, GPR35/CXCR8 has not been crystalized; however, GPR35/CXCR8 does contain many of the highly conserved Class A residue patterns in TMHs 1, 2, 3, 4, 5 (N1.50, D2.50, and (E)DRY motif in TMH3, C3.25, W4.50, and P5.50); see **Figure 1**. Interestingly, there are a few notable conserved motif differences: 1) a conservative substitution (CFLP) for the TMH6 CWXP motif, and 3) a non-conservative substitution (DAICY) for the TMH7 NPXXY motif. In addition, the GPR35/CXCR8 EC-1 (EC-1) loop is shorter than most [2 amino acids (aa) vs. 6 aa in β_2_-AR and Rho] and the GPR35/CXCR8 EC-3 loop is noticeably longer than most (11 aa long vs. 5 aa in β_2_-AR, 6 aa in Rho and CB1/CB2).

### Disulfide Bridge Positions

Like most Class A GPCRs, GPR35/CXCR8 also has a cysteine in the EC2 loop (C162) that can form a disulfide bridge with C3.25(89) (CB1 and CB2 are exceptions) and like CXCR4, GPR35/CXCR8 has key cysteines in the N-terminus [C(8)] and at the TMH7 EC end [C7.25(248)], that likely form another disulfide bridge.

### Intracellular Ionic Lock

TMH6 in most GPCRs has a negatively charged glutamate or aspartate in position 6.30. This residue interacts with R3.50 of the conserved DRY motif to form an “ionic lock” that keeps the IC end of the receptor closed and therefore inaccessible to G-protein. GPR35/CXCR8 does not have a negatively charged residue at 6.30, but has a threonine that can still form a hydrogen bond with R3.50 to keep the receptor IC domain closed.

### Model-Guided Studies

Several mutation and computational studies have probed GPR35/CXCR8’s structure, allowing for the identification of functionally important residues—ultimately informing novel ligand design. First, it has been reported that TMH3 (specifically Y3.32 and R3.36) is important for both human and rat GPR35/CXCR8 signal transduction (Jenkins et al., 2010). R3.36 was probed due to its importance in the binding of anionic ligands in GPR81 (Liu et al., 2009); in analogy, the authors hypothesized R3.36 may be important to GPR35/CXCR8’s ability to bind anionic ligands, such as kynurenic acid (**12**) (Jenkins et al., 2011). The authors reported that mutation of R3.36 or Y3.32 to an alanine abolished the ability of all tested agonists [including kynurenic acid (**12**) and zaprinast (**2**)] to activate human or rat GPR35/CXCR8; importantly, this mutation did not significantly impact global receptor expression and only significantly reduced rat R3.36A surface expression (though other mutants trended toward reduced surface expression; Jenkins et al., 2011). Additionally, Y3.32 was mutated to a leucine—at this mutant, tested agonists were able to signal (albeit with a significantly increased EC_50_) at rat GPR35/CXCR8, but signaling was completely abolished at human GPR35/CXCR8 (Jenkins et al., 2011). Altogether, the authors state that the similar functional outcomes of the tested agonists suggest (but does not prove) that the ligands may share a common orthosteric binding site, and that these mutation results suggest that R3.36 and Y3.32 may be key binding site residues (Jenkins et al., 2011).

In a later report, mutation and modeling studies were performed to investigate the function and binding site of several anti-allergy compounds at both human and rat GPR35/CXCR8 (MacKenzie et al., 2014). These compounds were tested at several human and mouse GPR35/CXCR8 mutants; mutation consisted of several species residue switches (human→rat, and *vice versa*) of positively charged residues hypothesized to be part of the orthosteric binding site. These mutations include, at human GPR35/CXCR8: R164S, R4.60M, L4.62R, R6.58Q, R7.32S, and R6.58Q-R7.32S; the reciprocal rat→human mutations were performed as well. Their results suggest that the impact of these mutations (at both human and rat GPR35/CXCR8) are highly ligand-specific; additionally, all of these mutations impacted at least two of the tested ligands, suggesting that these residues may be part of GPR35/CXCR8’s orthosteric binding site (with the exception of the rat→human Q6.58R, which appeared to have no effect on any of the agonists tested). Furthermore, the authors report that the mutation results were consistent with presented homology models of both human and rat GPR35/CXCR8; these models were also used to predict a detrimental human SNP. The SNP (V2.60M) was experimentally characterized and agonists were indeed found to have a reduced potency at this mutant. Altogether, these results provide valuable structural insight into GPR35/CXCR8’s binding site (especially regarding species selectivity).

**FIGURE 5:**
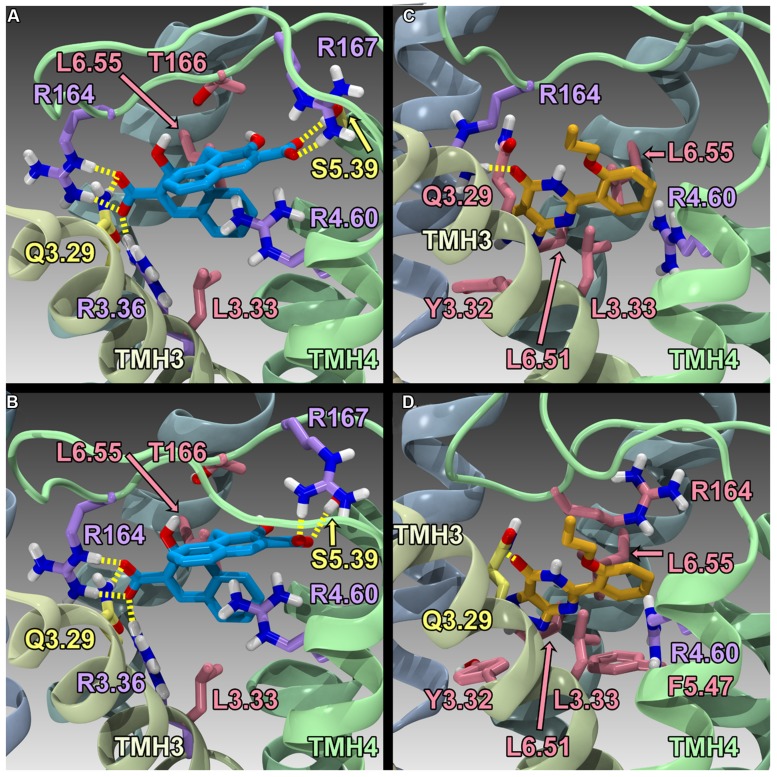
**Homology models of pamoic acid and zaprinast docked at WT and R6.58(240)A**. The view is from the lipid bilayer, toward TMH3-4; pamoic acid is shown in blue; zaprinast is shown in orange; residues that form a salt bridge/π interactions are shown in lavender; residues that form hydrogen bonds are shown in yellow; residues that form van der Waals interactions are shown in pink. **(A)** Pamoic acid docked in the human WT GPR35/CXCR8 model; **(B)** Pamoic acid docked in the human R6.58(240)A GPR35/CXCR8 model; **(C)** Zaprinast docked in the human WT GPR35/CXCR8 model; **(D)** Zaprinast docked in the human R6.58(240)A GPR35/CXCR8 model.

Finally, Zhao et al. (2014) have reported the results of human GPR35/CXCR8 mutation and computational studies. Homology models of human WT GPR35/CXCR8 in an inactive (R) and active (R*) state were constructed, as well as an R* model of R6.58(240)A GPR35/CXCR8. These models used the β_2_-adrenergeic receptor (β_2_-AR) crystal structure as an initial template (Cherezov et al., 2007); however, models were refined (to account for sequence differences between β_2_-AR and GPR35/CXCR8) using the Monte Carlo/simulated annealing technique Conformational Memories. Zaprinast (**2**) and pamoic acid (**1**) were manually docked in the final GPR35/CXCR8 models; these docks were refined with the automatic docking program, Glide. Both manual and automatic docking used Y3.32 and R3.36 as primary interaction sites. GPR35/CXCR8-ligand models (see **Figure 5**) were energy minimized and used to direct mutation studies: K1.32(20)A, R2.65(81)A, R3.36(100)A, A4.59(150)G, R4.60(151)A, R164A/L, R167A, R6.58(240)A, R7.33(256)A, and K7.40(263)A. Consistent with computational results, residues within the TMH1-2-7 region had no effect on ligand efficacies; likewise, residues in the TMH3-4-5-6 region were found to be important for agonist efficacy. Specifically, the models predicted favorable electrostatic interactions between the agonists and R164, as well as a favorable interaction between R167 and pamoic acid (**1**); consistently, mutation of these residues to alanine resulted in a loss in efficacy. Interestingly, the R4.60(151)A mutation resulted in a complete loss in signaling. In addition, the R6.58(240)A mutation did not significantly impact pamoic acid (**1**)’s ability to signal via GPR35/CXCR8; this is consistent with the reported computational results, as pamoic acid (**1**) did not form a significant interaction with R6.58(240)A in the WT or mutant receptor models. Finally, the R6.58(240)A mutation resulted in a 30 fold increase in zaprinast’s (**2**) potency; the models rationalize this by illustrating that R6.58(240) introduces steric crowding and that zaprinast (**2**) is able to form more favorable interactions when it is mutated to an alanine (see **Figure 5**). Together, these results suggest that zaprinast (**2**) and pamoic acid (**1**) bind the TMH3-4-5-6 region of GPR35/CXCR8, though each ligand forms unique interactions within its binding site.

## GPR55

**FIGURE 6:**
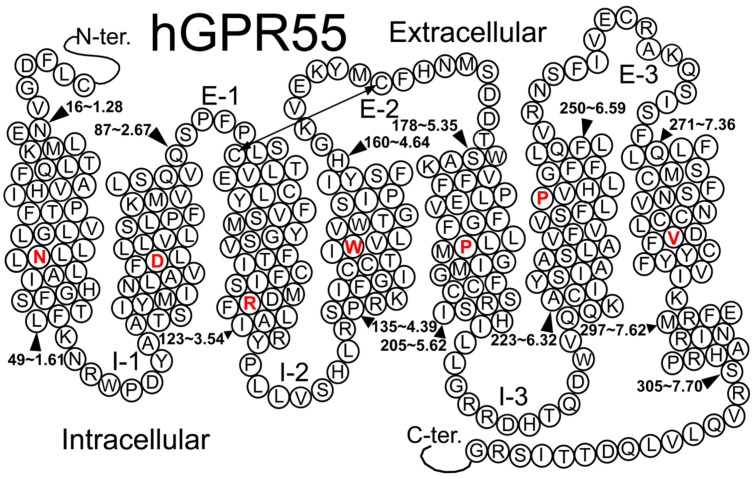
**A helix net representation for GPR55 is provided here**.

GPR55 belongs to the rhodopsin-like (Class A) family of GPCRs (Genbank accession # NM-005683; see helix net sequence representation in **Figure 6**). GPR55 was de-orphanized as a cannabinoid receptor (Brown and Wise, 2001; Drmota et al., 2004). It has the highest amino acid identity to the following receptors: GPR35 (27%), P2Y (29%), GPR23 (30%), and CXCR4 (26%; Sawzdargo et al., 1999). GPR55 exhibits lower amino acid identity to the cannabinoid CB1 (13.5%) and CB2 (14.4%) receptors.

### Signaling

GPR55 has been reported to couple to Gα13 (Ryberg et al., 2007; Henstridge et al., 2009), Gα12, or Gαq (Lauckner et al., 2008) proteins.

Activation of GPR55 also results in activation of PLC, RhoA, ROCK, ERK, p38 mitogen activated protein kinase, and Ca^2+^ release that can induce downstream transcription factors such as NFAT, NF-κB, CREB, and ATF2 (Lauckner et al., 2008; Henstridge et al., 2009, 2010; Kapur et al., 2009; Oka et al., 2010a). It has recently also been shown that GPCR-associated sorting protein 1 (GASP-1) is an important regulator of ligand-mediated down-regulation of GPR55 (Kargl et al., 2012).

## Cannabinoid Ligands Recognized at GPR55

**FIGURE 7:**
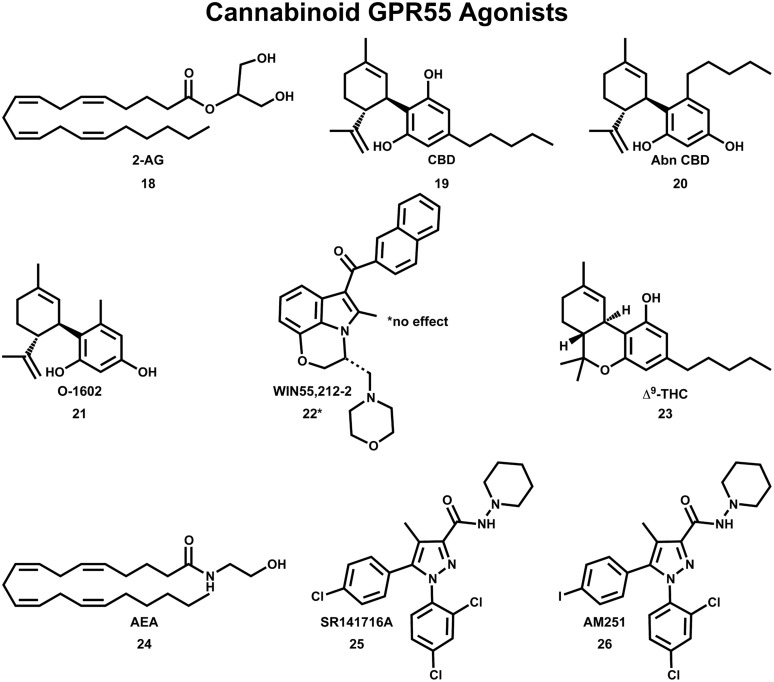
**Cannabinoid agonists that activate GPR55 are shown here**.

The initial deorphanization of GPR55 as a cannabinoid receptor spurred a wide search for GPR55 ligands among known cannabinoid ligands (Henstridge et al., 2009). Initial studies confirmed that various cannabinoid and atypical cannabinoid compounds activate GPR55 (Johns et al., 2007; Ryberg et al., 2007; Lauckner et al., 2008). However, some of these studies did not agree concerning the pharmacological action for the same cannabinoid compound at GPR55. GTPγS functional assays indicated that GPR55 is activated by nanomolar concentrations of the endocannabinoids 2-AG **(18)**, virodhamine, noladin ether, oleoylethanolamide and palmitoylethanolamide and the atypical cannabinoids CBD **(19)** and abn-CBD **(20)**; see **Figure 7** (Ryberg et al., 2007). In a separate study, abn-CBD (**20**) and O-1602 (**21**) were found to act as agonists at GPR55, while the cannabinoid aminoalkylindole, WIN55,212-2 (**22**) produced no effect (Johns et al., 2007). Using an IC Ca^2+^ assay, Lauckner et al. (2008) reported that Δ^9^-THC (**23**), the aminoalkylindole, JWH-015, anandamide (AEA;**24**) and R-methanandamide acted as GPR55 agonists, while the CB1 antagonist, SR141716A (**25**), acted as a GPR55 antagonist. The CB1 antagonist, AM251 (**26**) was shown to elicit a GPR55-mediated Ca^2+^ signal in one study (Henstridge et al., 2009) and an increase in GTPγS binding in another study (Ryberg et al., 2007).

**FIGURE 8:**
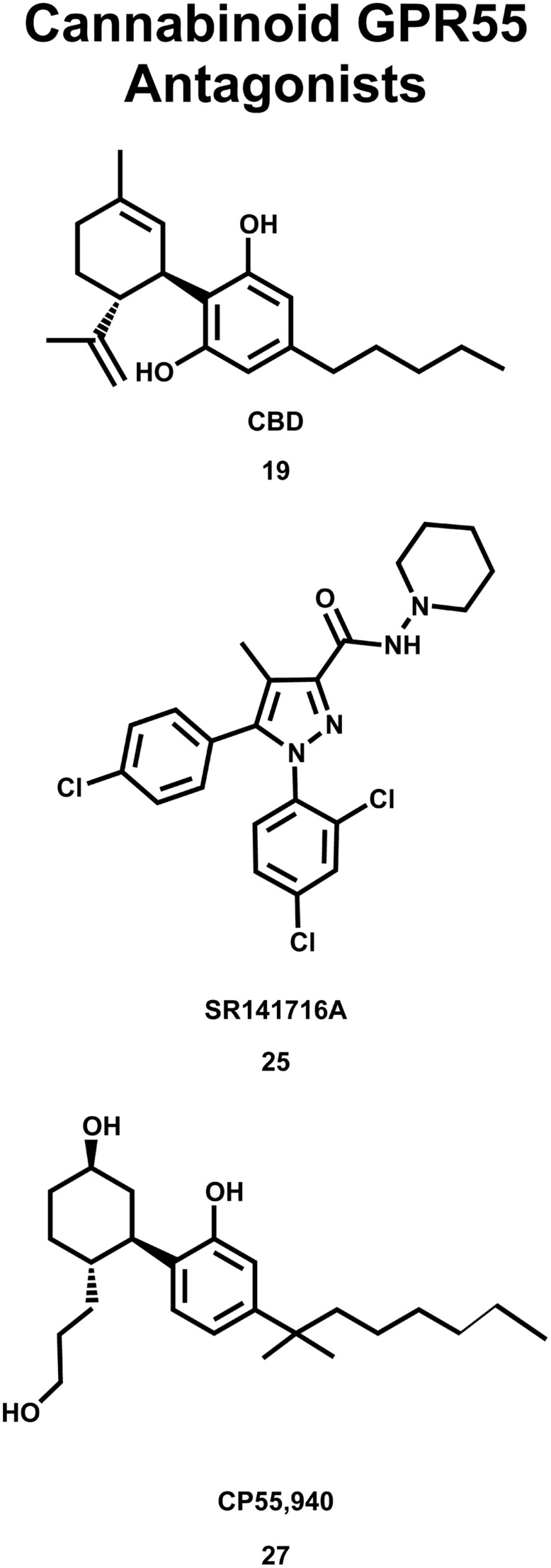
**Cannabinoid antagonists that inhibit GPR55 signaling are shown here**.

**FIGURE 9:**
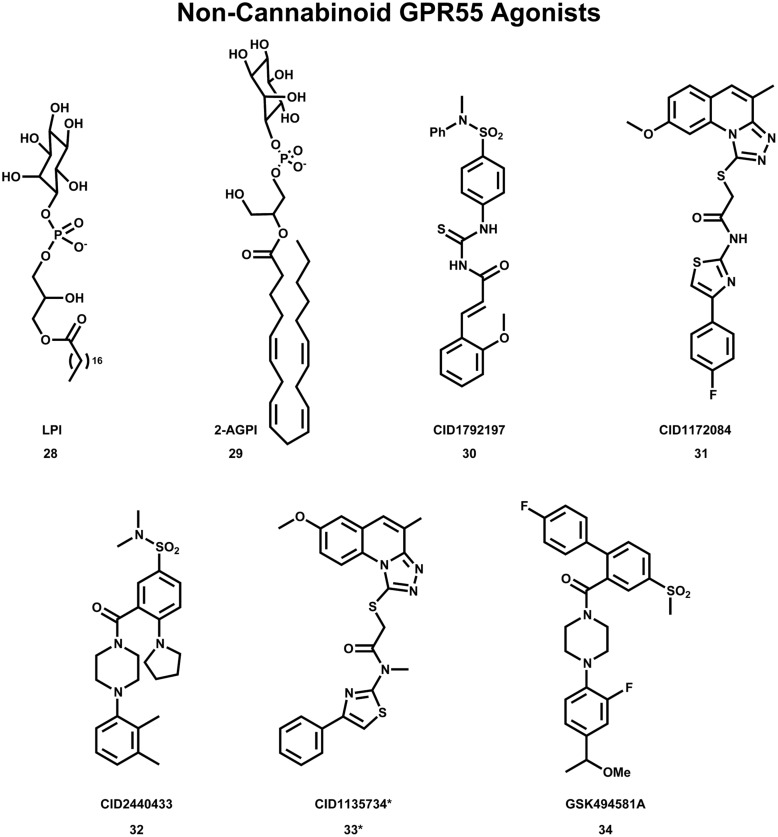
**Non-Cannabinoid agonists that activate GPR55 are shown here**. *CID1135734 (34), while structurally related to other GPR55 agonists, does not bind to GPR55 and was used as a negative control.

The deorphanization of GPR55 as a cannabinoid receptor has been controversial in the literature. Oka and co-workers reported that lysophosphatidylinositol (LPI, **28;** see **Figure 9**) compounds are endogenous GPR55 agonists (Oka et al., 2007), with 2-arachidonoyl-sn-glycero-3-phosphoinositol (2-AGPI; **29**) possessing the best LPI activity observed to date (Oka et al., 2009). Neither LPI nor 2-AGPI, however, bind to CB1 or CB2 receptors. Kapur et al. (2009) confirmed that LPI (**28**) is a GPR55 agonist in their screen for GPR55 ligands using a β-arrestin green fluorescent protein biosensor. These investigators also found that the cannabinoid CB1 antagonists AM251 (**26**) and SR141716A (**25**) were also GPR55 agonists (Kapur et al., 2009). These GPR55 ligands activated the G protein dependent signaling of PKCβII and possessed comparable efficacy in inducing β-arrestin trafficking. In contrast, the cannabinoid agonist CP55940 (**27**, **Figure 8**) acted as a GPR55 partial agonist/antagonist, producing the formation of β-arrestin GPR55 complexes, and the phosphorylation of ERK1/2, but inhibiting GPR55 internalization (Kapur et al., 2009). Ryberg et al. (2007) reported that CBD (**19**) was an agonist at GPR55, however, Whyte et al. (2009) found CBD (**19**) to function as a GPR55 antagonist. Here, CBD attenuated effects produced by GPR55 agonists O-1602 and LPI on human and mouse osteoclast polarization and resorption *in vitro*.

## Non-Cannabinoid GPR55 Agonists

Despite the many cannabinoid ligands identified to act at GPR55, no cannabinoid ligand has been found to have low nanomolar potency at GPR55. One reason for this is that initial ligand searches were conducted using cannabinoid receptor/lipid biased compound libraries and not by casting a wider net (Yin et al., 2009; Brown et al., 2011). For this reason, discovering and characterizing novel GPR55 chemotypes is still a crucial step in the GPR55 field. Results from wider screens are now appearing in the literature. Brown et al. (2011) used diversity screening to identify (1-{2-fluoro-4-[1-(methyloxy)ethyl]phenyl}-4{4′-fluoro-4-(methylsulfonyl)-2-biphenylyl]carbonyl} piperazine), GSK494581A (**34**) as a selective small-molecule ligand of GPR55. In collaboration with the Sanford-Burnham screening center of the Molecular Libraries Probe Production Centers Network (MLPCN), the Abood laboratory used a high content, high throughput β-arrestin screen (see mli.nih.gov/mli/mlp-probes/) to identify a series of GPR55 agonists that belong to novel, GPR55 agonist chemotypes (Heynen-Genel et al., 2010). The structures of three of these novel agonists **30** (EC_50_ = 0.11 μM), **31** (EC_50_ = 0.16 μM) and **32** (EC_50_ = 0.26 μM) are illustrated in **Figure 9**. The goal of the Abood lab is to use these agonists to design second generation, nanomolar efficacy ligands.

## Non-Cannabinoid GPR55 Antagonists

**FIGURE 10:**
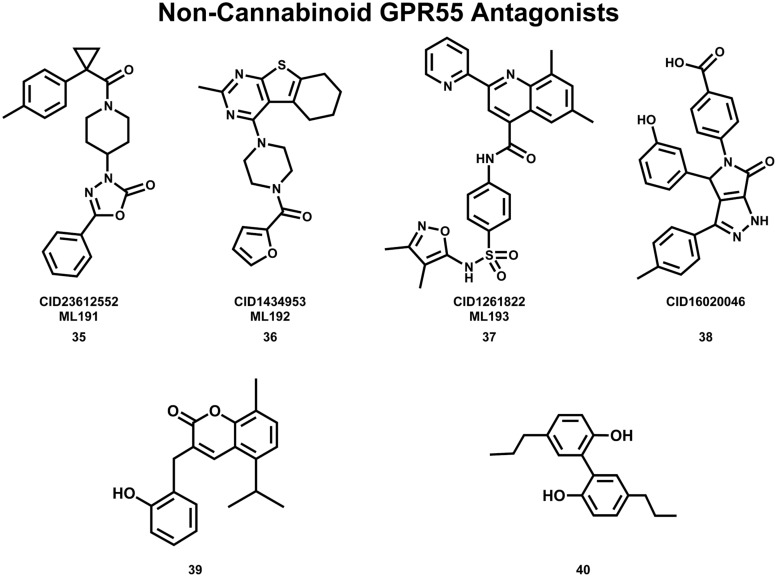
**Non-Cannabinoid antagonists that inhibit GPR55 signaling are shown here**.

The Abood lab, in collaboration with the Sanford-Burnham screening center of the MLPCN, also has identified new, non-cannabinoid GPR55 antagonists using a β-arrestin, high-throughput, high-content screen of ∼300,000 compounds. This screen yielded novel, GPR55 antagonist chemotypes with IC_50_s in the 0.16–2.72 μM range, many of which being completely selective, with no observed agonism or antagonism against GPR35, CB1 or CB2 up to 20 μM (Heynen-Genel et al., 2010 “Screening for Selective Ligands for GPR55 – Antagonists” [ML191, ML192, ML193] Bookshelf ID: NBK66153; PMID: 22091481). Three GPR55 antagonists identified in this screen were nominated as probe compounds for future studies. These are ML191 (CID23612552) (**35**), ML192 (CID1434953) (**36**), and ML193 (CID1261822) (**37)** (see **Figure 10** for compound drawings).

Additional GPR55 antagonists have been reported by other laboratories. CID16020046 (**38**) (Kargl et al., 2013) and 3-(2-hydroxybenzyl)-5-isopropyl-8-methyl-2**H**-chromen-2-one (**39**) (Rempel et al., 2013b) are GPR55 selective; while CBD (**19**) (Whyte et al., 2009; McHugh et al., 2010) and tetrahydromagnolol (**40**) (Rempel et al., 2013a) act at additional receptors.

## GPR55 Sequence Analysis and Important Features

**Figure 6** illustrates a helix net representation of the GPR55 amino acid sequence. GPR55 possesses most of the conserved Class A patterns in transmembrane helices (TMHs) 1, 2, 4, and 5 (N1.50, D2.50, W4.50, and P5.50), with the following conserved motif differences: (1) the TMH3 D/ERY motif is substituted conservatively with DRF, (2) the TMH6 CWXP hinge motif is substituted conservatively with SFLP, and (3) the TMH7 NPXXY motif has a non-conservative substitution, DVFCY. There are also important loop length differences. These are notable because the distance that the ends of helices can be apart from one another is limited by the length of the loops connecting them. The GPR55 extracellular-1 (EC-1) loop is shorter than most (3 aa vs. 6 aa in β_2_-AR and Rho) and the GPR55 EC-3 loop is noticeably longer than most (14 aa long vs. 5 aa in β_2_-AR, 6 aa in Rho and CB1/CB2).

### Disulfide Bridge Positions

GPR55 has a cysteine in the extracellular 2 (EC2) loop (C168) that can form a disulfide bridge with C3.25(94). This feature is seen in most Class A GPCRs with CB1 and CB2 being notable exceptions. The GPR55 sequence suggests a second disulfide bridge seen in the CXCR4 crystal structure (Wu et al., 2010), GPR55 has cysteines in the N-terminus [C(10)] and at the TMH7 (EC) end (C7.25), that likely form this second disulfide bridge.

### Intracellular Ionic Lock

In most Class A GPCRs, residue 6.30 at the IC end of TMH6 is negatively charged (D/E6.30). This residue forms a salt bridge (called the “ionic lock”) with R3.50 of the D/ERY motif at the IC end of TMH3. This “ionic lock” keeps the IC end of the receptor closed and therefore inaccessible to G protein. Although GPR55 does not have a negatively charged residue at 6.30, it has a glutamine. Q6.30 can still form a hydrogen bond with R3.50 to keep the receptor IC domain closed.

### Binding Pocket Toggle Switch

G protein-coupled receptors also have a set of residues within the binding pocket that act as a “toggle switch” which controls the transition form the inactive state (R) to the activated state (R*). This toggle switch involves a residue on TMH6, 6.48 [F6.48(239) in GPR55] whose change from a χ^1^ torsion angle of *g+* to *trans* causes a straightening of TMH6 at its IC end that breaks the IC “ionic lock.” In the inactive state, the χ^1^ of F6.48 is kept in *g+* via an interaction with another residue. For GPR55, this residue is M3.36(104). Antagonists will stabilize the interaction between M3.36(104) and F6.48(239). Agonists prefer a binding pocket in which the toggle switch residues have undergone torsion angle changes that move these residues away from each other [F6.48 χ^1^ *g+* →*trans; M3.36(104) trans*→* g+*] permitting TMH6 to straighten.

### Primary Ligand Interaction Site

The CB1 and CB2 receptors have a single positively charged residue within the TMH domain, K3.28. K3.28 in CB1 has been shown to be the primary ligand interaction site for classical, non-classical and endo-cannabinoids, as well as, for the biarylpyrazole inverse agonists (Song and Bonner, 1996; Hurst et al., 2002). GPR55 has one positively charged residue, K2.60, which mutation studies indicate to be important for ligand binding (Kotsikorou et al., 2011). For this reason, K2.60 has been used as a primary interaction site for docking studies of ligands at GPR55 (Kotsikorou et al., 2011, 2013).

### GPR55 R and R* Models

Models of the GPR55 inactive (R) and activated (R*) states have been published that explored the GPR55 agonist (Kotsikorou et al., 2011) and antagonist binding sites at GPR55 (Kotsikorou et al., 2013). These models were based initially upon the 2.4Å crystal structure of the β_2_-AR (PDB Name: 2RH1; Cherezov et al., 2007) and then modified to reflect sequence dictated conformational differences in TMHs 1,2,5,6 and 7 [please see a complete discussion in the paper (Kotsikorou et al., 2011)]. Because GPR55 has considerable sequence similarity with the CXCR4 receptor (26%; Sawzdargo et al., 1999) and because their sequences share a key second disulfide bridge, involving N-terminus residue [C(10) in GPR55; C(28) in CXCR4] and TMH7 residue (C7.25 in both GPR55 and CXCR4), the current GPR55 model in the Reggio lab has been updated to include an N-terminus/TMH7 disulfide bridge by analogy with the CXCR4 crystal structure (PDB Name: 3ODU; Wu et al., 2010). This N-terminus/TMH7 disulfide bridge helps to open up the EC region of the receptor.

## GPR55 Agonist Binding Studies

**FIGURE 11:**
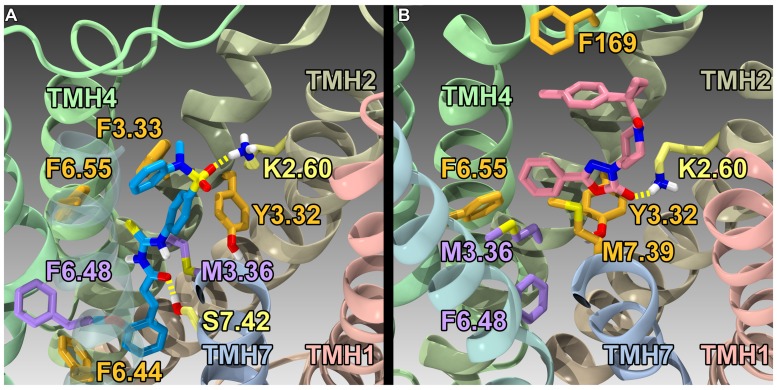
Homology models of CID1792197 (**30**; agonist) and ML191 (**35**; antagonist) docked at WT R* (active) and R (inactive) GPR55 models, respectively. The view is from the lipid bilayer, toward TMH6-7; residues that form the ‘toggle switch’ are shown in lavender; residues that form hydrogen bonds are shown in yellow; residues that form van der Waals interactions are shown in orange; hydrogen bonds are shown as dashed yellow lines. **(A)** CID1792197 (agonist, blue) in the human WT GPR55 R* model; **(B)** ML191 (antagonist, pink) in the human GPR55 R model. Notice that ML191 is docked more extracellularly than CID1792197 and sterically blocks the toggle switch residues (lavender) from undergoing necessary conformational changes via interactions with F6.55 (i.e., ML191 packs against F6.55, which in turn packs against M3.36).

Using the GPR55 model for the activated state (R*), Kotsikorou et al. (2011) studied the binding of a series of GPR55 agonists: LPI (**28**) and three novel agonists obtained from the Sanford-Burnham screen, **30** (EC_50_ = 0.11 μM), **31** (EC_50_ = 0.16 μM) and **32** (EC_50_ = 0.26 μM). These structures are shown with PubChem Compound IDs in **Figure 9** (Kotsikorou et al., 2011). Closely related compound **33** (EC_50_ > 32 μM), that does not bind to GPR55 served as a negative control. Similarities in shapes, as well as molecular electrostic potential (MEP) similarities were identified. Modeling data indicated that the similarity between **30**, **31**, **32,** and LPI (**28**) enables them all to be recognized by a single GPR55 binding pocket. The shape of the GPR55 R* binding site accommodates ligands that are inverted-L shapes or T shapes with long, thin profiles that can fit vertically deep in the receptor binding pocket, while their broad head regions occupy the horizontal binding pocket opening near the EC loops. The vertical pore is narrow enough that it cannot accommodate the *N*-methyl group of **33** (negative control). For GPR55 agonist ligands (**30–32**), the most negative electrostatic potential region is exposed either at the “elbow” of the L or at one end of the T cross bar [see red regions in **Figure 5** in the paper (Kotsikorou et al., 2011)]. It is this region that interacts with K2.60 in each of the docks. **Figure 11A** shows CID1792197 (**30**; blue) docked in the WT R* GPR55 model. The view is from the lipid bilayer, toward TMH6-7; residues that form the ‘toggle switch’ are shown in lavender; residues that form hydrogen bonds are shown in yellow; residues that form van der Waals interactions are shown in orange; hydrogen bonds are shown as dashed yellow lines.

Other reported GPR55 agonists have been docked in this GPR55 R* model to test the model. The CB1 antagonist/GPR55 agonist, AM251 (**26**) (Kapur et al., 2009) adopts a T-shape, with its pyrazole and 2,4-dichlorophenyl rings forming the cross bar of this T-shape and the 4-iodophenyl ring extending into the vertical section of the binding pocket. The structure of benzoylpiperazine GPR55 agonist, (GSK494581A; **34**) (Brown et al., 2011) is similar to **32**. **34** adopts a T-shape at GPR55 R*, binding in the same receptor region and in a similar orientation as **32** with comparable energy of interaction [see Supplementary Information in (Kotsikorou et al., 2011)].

## GPR55 Antagonist Binding Studies

Using a model of GPR55 inactive state, Kotsikorou et al. (2013) studied the binding of an antagonist series that emerged from the Sanford-Burnham screen, ML191 (**35**), ML192 (**36**) and ML193 (**37**). These studies suggested that GPR55 antagonists possess a head region that occupies a horizontal binding pocket extending into the EC loop region, a central ligand portion that fits vertically in the receptor binding pocket and terminates with a pendant aromatic or heterocyclic ring that juts out. Both the region that extends extracellularly and the pendant ring are features associated with antagonism (see below). **Figure 11B** illustrates ML191 (**35**; pink) docked in the WT R (inactive) GPR55 model. The view is from the lipid bilayer, toward TMH6-7; residues that form the ‘toggle switch’ are shown in lavender; residues that form hydrogen bonds are shown in yellow; residues that form van der Waals interactions are shown in orange; hydrogen bonds are shown as dashed yellow lines. This figure shows that ML191 (**35)** is docked more extracellularly compared with the agonist CID1792197 (**30**) in the R* model (see **Figure 11A**). In this position, the pendant phenyl ring of ML191 (**35)** sterically blocks the toggle switch residues (lavender) from undergoing necessary conformational changes via interactions with F6.55 (i.e., ML191 (**35)** packs against F6.55, which in turn packs against M3.36).

Docks of two other recently reported GPR55-selective antagonists CID16020046 (**38**) (Kargl et al., 2013) and 3-(2-hydroxybenzyl)-5-isopropyl-8-methyl-2**H**-chromen-2-one (**39**) (Rempel et al., 2013b) show that these two compounds also adopt a similar orientation at GPR55 (Kotsikorou et al., 2013). While two other GPR55 antagonists that are non-selective, tetrahydromagnolol (**40**) (Rempel et al., 2013a) and CBD (**19**) (Whyte et al., 2009; McHugh et al., 2010) do not completely conform to the general shape description. The fact that these are also the only two ligands that lack GPR55 selectivity leads to the speculation that this shape difference may be the origin of their non-selectivity (Kotsikorou et al., 2013).

## The Key Molecular Features that Discriminate GPR55 Antagonists from Agonists

There are two striking differences between the GPR55 antagonist vs. agonist structures. GPR55 antagonists possess a head region that occupies a horizontal binding pocket extending into the EC loop region, a central ligand portion that fits vertically in the receptor binding pocket and terminates with a pendant aromatic or heterocyclic ring that juts out. This pendant portion in ML193 (**37**), ML192 (**36**) and ML191 (**35**) may be able to prevent putative toggle switch residues, M3.36(104)/F6.48(239) from undergoing any conformational change by sterically blocking their movement. Second, the location of the most negative electrostatic potential region of the two classes (antagonist vs. agonist) deviate from each other. The agonists have their most electronegative region [which hydrogen bonds to K2.60(80)] near the broad head region, while the most electronegative region found in antagonists is near the end of the central portion of the molecule. Because K2.60(80) is located two turns from the EC end of the GPR55 TMH bundle, the GPR55 antagonists bind higher than GPR55 agonists, extending into the EC loop region. The EC-2 loop residue, F169, for example is an important interaction site for ML191 (**35**), ML192 (**36**), and ML193 (**37**). EC-2 loop conformational changes have been reported to be critical for signal transduction of numerous Class A GPCRs (Unal et al., 2010). The fact that ML191 (**35**), ML192 (**36**), and ML193 (**37**) all bind high enough in GPR55 to interact directly with the EC-2 loop and block its movement may be another reason for the antagonism exhibited by these compounds.

On the other hand, GPR55 agonist structures lack the pendant phenyl or heterocyclic ring of the antagonists that juts out after the vertical central portion. It is this portion of the antagonists that stabilizes the toggle switch in its “off” conformation. Instead, agonist structures maintain a thin vertical profile in the binding pocket that relies on the M3.36(104)/F6.48(239) toggle switch residues having undergone the [F6.48 χ^1^ *g+* →*trans; M3.36(104) trans*→* g+*] transition to provide room for the agonist to penetrate deep into the binding pocket.

## GPR55 Expression Profile and Therapeutic Potential

GPR55 has been shown to be expressed in numerous tissues throughout the body, in mouse, rat and human tissues (Sawzdargo et al., 1999; Ryberg et al., 2007; Moreno-Navarrete et al., 2012). In the human CNS, GPR55 is found predominantly in the caudate, putamen, and striatum (Sawzdargo et al., 1999). In mice, GPR55 mRNA is most abundantly expressed in the adrenal tissue, ileum, jejunum, frontal cortex and striatum (Ryberg et al., 2007). Numerous studies have indicated that GPR55 activation is pro-carcinogenic (Ford et al., 2010; Andradas et al., 2011; Pineiro et al., 2011). Bone cells including osteoblasts and osteoclasts have been found to also express GPR55 (Abed et al., 2009; Rossi et al., 2009; Whyte et al., 2009).

In addition, GPR55 is expressed in tissue that is involved in regulating energy intake and expenditure. Localization studies in different organisms have shown that this receptor has been identified in the hypothalamus in mice (Ryberg et al., 2007) and in different regions of the gastrointestinal tract, including the esophagus, stomach, jejunum and colon in mouse (Ryberg et al., 2007), and jejunum, ileum and colon in rat (Lin et al., 2011; Schicho et al., 2011). In the rat small intestine, GPR55 was localized mainly in the submucosa and myenteric plexus (Lin et al., 2011). GPR55 mRNA and protein expression have also been located in the liver in rats (Sawzdargo et al., 1999), mice (Romero-Zerbo et al., 2011), and humans (Moreno-Navarrete et al., 2012), in adipose tissue from rats (Romero-Zerbo et al., 2011), in visceral and subcutaneous white adipose tissue (WAT) in humans (Moreno-Navarrete et al., 2012) and in pancreas from rat, specifically the islets of Langerhans (Romero-Zerbo et al., 2011). GPR55 tissue expression in brown adipose tissue has yet to be determined. Importantly, the localization of GPR55 in several tissues involved in regulating energy intake and expenditure suggests a role for this GPCR in the maintenance of energy homeostasis.

Based upon this expression pattern, there are at least three therapeutic areas in which GPR55 may prove useful: (1) the regulation of energy intake and expenditure, which impacts the fields of obesity and diabetes (Simcocks et al., 2014; Liu et al., 2015); (2) resorption of bone, which impacts the field of osteoporosis (Whyte et al., 2009); and (3) agonist pro-carcinogensis, which impacts many types of cancers (Ford et al., 2010; Andradas et al., 2011; Pineiro et al., 2011; Leyva-Illades and Demorrow, 2013). These categories are discussed below.

### Obesity and Type-2 Diabetes

The first reports of biological activity for LPI suggested that LPI is involved in stimulation of insulin release from pancreatic islets via mobilization of Ca^2+^ ions (Metz, 1986a,b). These results suggest that LPI may play a role in whole body metabolism, as well as in obesity and type-2 diabetes. The GPR55 agonist, O-1602 (**21**), has also been shown to influence obesity, because this compound increased food intake and adiposity in Sprague-Dawley rats (Diaz-Arteaga et al., 2012). However, the increase in food intake was still evident in GPR55^-/-^ mice (Diaz-Arteaga et al., 2012), indicating that this compound was also acting on other receptor(s). There is increasing evidence that GPR55 may play a role in homeostatis as well. A link between a GPR55 gene polymorphism and anorexia nervosa has been reported (Ishiguro et al., 2011). GPR55 has been shown to be expressed in human visceral and subcutaneous adipose tissue (VAT and SAT), as well as in liver (Moreno-Navarrete et al., 2012). Moreno-Navarrete et al. (2012) and co-workers reported that GPR55 expression in VAT is positively associated with obesity and type-2 diabetes. LPI plasma levels were found to be higher in obese compared to lean patients. In differentiated adipocytes from visceral fat of obese patients, LPI raised IC calcium levels. These results suggested that the LPI/GPR55 system is positively associated with obesity in humans (Moreno-Navarrete et al., 2012). Taken together, these studies would suggest that a GPR55 agonist may increase weight gain and fat storage (Henstridge, 2012).

Most recently, Imbernon et al. (2014) have investigated the regulation of GPR55 in rat WAT in different physiological and pathophysiological settings involved in energy balance. They compared GPR55 expression with CB1 receptor expression by real time PCR and western blotting. Circulating levels of LPI (**28**) were measured by liquid chromatography-mass spectrometry. Both WAT CB1 and GPR55 levels were increased after fasting and recovered after leptin treatment. Their expression was decreased during gestation and increased throughout lifespan. Orchidectomy diminished WAT CB1 and GPR55 expression, whereas ovariectomized rats showed increased GPR55 but decreased CB1 levels. Alterations in pituitary functions also modified WAT CB1 and GPR55 levels. However, serum LPI levels were inversely regulated by fasting and gonadectomy in comparison to WAT GPR55. These results suggest that GPR55 and LPI are regulated by different physiological and patho-physiological settings known to be associated with marked alterations in energy status (Imbernon et al., 2014).

Understanding the role of GPR55 in energy homeostasis may provide a novel target for therapeutic intervention in type-2 diabetes (Liu et al., 2015). High GPR55 mRNA expression has been found in pancreatic islets and protein expression was found in insulin-secreting β-cells (Romero-Zerbo et al., 2011). GPR55 is expressed in β cells, which secrete insulin, whereas neither α cells, which secrete glucagon, nor δ cells, which secrete somatostatin (Elayat et al., 1995), express GPR55 (Romero-Zerbo et al., 2011). This cellular localization suggests involvement of GPR55 in the endocrine function of the pancreas, but only through insulin secretion and possibly the maintenance of blood glucose levels (Romero-Zerbo et al., 2011). O-1602 (**21**) activation of GPR55 produced an increase in Ca^2+^ release and insulin secretion stimulated by glucose. The latter was reduced in GPR55^-/-^ mice. Further *in vivo* experiments showed that GPR55 activation increases glucose tolerance and plasma insulin levels. McKillop et al. (2013) assessed the effects of various GPR55 agonists on glucose homeostasis. GPR55 expression in pancreatic β cells was confirmed and GPR55 was demonstrated to be a strong activator of insulin secretion, with glucose lowering effects *in vivo*.

### Bone

Bone cells including osteoblasts and osteoclasts express GPR55 (Abed et al., 2009; Rossi et al., 2009; Whyte et al., 2009). LPI has been proposed to play an important role in bone physiology due to regulation of osteoclast number and function. Whyte et al. (2009) confirmed a high level of GPR55 expression in murine and human osteoclasts, implying involvement of LPI in stimulation of osteoclast polarization and bone resorption.

Whyte et al. (2009) also showed that LPI and O-1602 both inhibited osteoclast formation from bone marrow macrophages *in vitro*. The GPR55 antagonist, CBD increased osteoclast formation. GPR55 agonists stimulated the resorptive activity of osteoclasts, although they were found to inhibit osteoclast formation. CBD enhanced osteoclast formation and inhibited resorptive activity. These observations suggest that GPR55 activation inhibits osteoclast formation, but increases the ability of osteoclasts to resorb bone. GPR55 antagonism increases osteoclast formation, but reduces the ability of osteoclasts to resorb bone. In GPR55**^-/-^** mice, Whyte et al. (2009) found a sex based difference. Male GPR55**^-/-^** mice, had high peak bone mass affecting the trabecular compartment of the tibia and femur. Impairment of bone resporption appears to be the mechanism, although the reasons responsible for the differences in skeletal phenotype in these animals is unclear.

In regenerative processes such as bone healing, migration and differentiation of mesenchymal stem cells (MSCs) are known to be involved. However, little is known about the pharmacotherapeutic options aiming at the mobilization and differentiation of MSCs. Schmuhl et al. (2014) recently demonstrated that CBD promotes the migration of MSCs via activation of the CB2 receptor and inhibition of GPR55. CBD also induces osteoblastic differentiation. CBD may therefore recruit MSCs to sites of calcifying tissue regeneration and subsequently support bone regeneration via an osteoanabolic action on MSCs (Schmuhl et al., 2014).

### Cancer

Ruban et al. (2014) recently reported a link between LPI and ATP-binding cassette (ABC) transporter C1 (ABCC1)/multidrug resistance protein 1 (MRP1). These investigators discovered that LPI synthesized by cytosolic phospholipase A2 (cPLA2) is pumped out of the cell by ABC transporter C1 (ABCC1)/(MRP1), initiating a signaling cascade downstream of GPR55. These results suggest that blockade of this pathway may represent a novel strategy to inhibit cancer cell proliferation (Ruban et al., 2014).

The GPR55 endogenous ligand, LPI is secreted by fibroblasts and epithelial cancer cells and transformed thyroid cells, leading to mitogenic effects (Falasca and Corda, 1994; Falasca et al., 1998). Increased LPI plasma levels have been found in ovarian cancer patients compared with healthy control patients (Xiao et al., 2000). Based upon knowledge of such links between LPI and cancer, numerous studies have explored the link between GPR55 and cancer.

### Skin Cancer

Activation of GPR55 has been shown to enhance skin cancer cell anchorage-independent growth, invasiveness and tumorigenicity *in vivo* (Pérez-Gómez et al., 2013). This suggests that GPR55 promotes tumor growth and aggressiveness. Pérez-Gómez et al. (2013) have shown that GPR55**^-/-^** mice are more resistant to DMBA/TPA-induced papilloma and carcinoma formation than their wild-type littermates (Pérez-Gómez et al., 2013). In human skin tumors and squamous cell carcinomas, GPR55 is also upregulated compared with GPR55 levels in healthy tissues. Thus, GPR55 appears to be pivotal in skin tumor development. This suggests not only that GPR55 antagonists may have therapeutic value in skin cancer, but also that this receptor could be used as a new biomarker in squamous cell carcinomas (Pérez-Gómez et al., 2013). Lymphoblastoid cell lines, human astrocytoma, melanoma, and B lymphoblastoma tumors have also been reported to have GPR55 expression (Oka et al., 2010a; Andradas et al., 2011).

### Breast Cancer

In response to the tumor microenvironment, LPI and GPR55 play a role in the modulation of migration, orientation and polarization of breast cancer cells (Ford et al., 2010). Ford et al. (2010) found GPR55 expression in the highly metastatic MDA-MB-231 human breast cancer cell line. This expression was less abundant in the less-metastatic human cell line, MCF-7 (Ford et al., 2010). MDA-MB-231 cell chemotaxis was enhanced by treatment with prevented. LPI treatment of MDA-MB-231 cells significantly enhanced cell chemotaxis. This effect could be prevented using GPR55 siRNA (Ford et al., 2010). Andradas et al. (2011) have found expression of GPR55 in human breast tumors. Higher levels of GPR55 were documented in tumors with the worst prognosis (Andradas et al., 2011). These investigators also observed an association between increased GPR55 levels and high proliferative indexes, but not tumor size or metastasis. Overexpression of GPR55 increased cell viability and ERK phosphorylation. Decreased cell viability and ERK phosphorylation were observed when GPR55 was downregulated. The proliferative effects mediated by GPR55 have been proposed to be the result of ERK activation and downstream expression of c-Fos (Andradas et al., 2011).

### Prostate and Ovarian Cancer

Pineiro et al. (2011) found that human ovarian (OVCAR3 and A2780) and prostate (PC-3 and DU145) cancer cell lines have GPR55 mRNA and protein expression. Treatment of these cells with LPI produced a transient increase in IC Ca^2+^, ERK and Protein Kinase B (Akt) phosphorylation. Treatment of these cells with GPR55 siRNA reversed these effects, suggesting that GPR55 may mediate LPI effects in ovarian and prostate cancer cells. Downregulation of GPR55 inhibited cancer cell proliferation without addition of exogenous LPI. This suggests that cancer cells may release LPI and promote proliferation in an autocrine loop via GPR55 (Pineiro et al., 2011).

### Cholangiocarcinomas

Neoplastic transformation of the epithelial cells that line the biliary ducts produces cholangiocarcinomas (DeMorrow et al., 2007). With the identification of GPR55 as a novel cannabinoid receptor capable of regulating the effects of AEA, Huang et al. (2011) showed that both malignant and non-malignant cholangiocytes express GPR55 to a similar degree. O-1602 had a suppressive effect on cholangiocarcinoma growth both *in vitro* and *in vivo* at a level similar to that of AEA. The antiproliferative action of AEA can be prevented by knocking down GPR55 expression. The growth-suppressing effects of GPR55 activation by AEA require Gα12 and Jun N-terminal kinase activation and subsequent translocation of Fas into the lipid raft structures. These data suggest that GPR55 offers an intriguing target for the design of potential chemotherapeutic agents directed at neoplastic transformation of bile duct epithethial cells (Leyva-Illades and Demorrow, 2013).

### Glioblastoma

Higher histological grade human glioblastomas have been reported to be associated with increased GPR55 expression. Silencing GPR55 in a xenograft model of glioblastoma slowed tumor growth and reduced the number of proliferation cells within the tumors (Andradas et al., 2011).

### Pancreatic Cancer

Increasingly advanced stages of human pancreatic ductal adenocarcinoma has been linked with high GPR55 levels (Andradas et al., 2011).

## Conclusion

It is hoped that this review will suggest to the GPR55 scientific community, additional ligands that can be used to study effects produced by GPR55. New, selective, non-cannabinoid GPR55 ligands have not found there way into published studies yet. Use of such ligands may resolve some of the ambiguities that currently exist in the GPR55 literature concerning effects produced by endogenous ligands such as LPI that may not be due to action at GPR55 (Drzazga et al., 2014).

## Conflict of Interest Statement

The authors declare that the research was conducted in the absence of any commercial or financial relationships that could be construed as a potential conflict of interest.
